# Insights into the Correlation and Immune Crosstalk Between COVID-19 and Sjögren’s Syndrome Keratoconjunctivitis Sicca via Weighted Gene Coexpression Network Analysis and Machine Learning

**DOI:** 10.3390/biomedicines13030579

**Published:** 2025-02-25

**Authors:** Yaqi Cheng, Liang Zhao, Huan Yu, Jiayi Lin, Meng Li, Huini Zhang, Haocheng Zhu, Huanhuan Cheng, Qunai Huang, Yingjie Liu, Tao Wang, Shiqi Ling

**Affiliations:** 1 Department of Ophthalmology, The Third Affiliated Hospital, Sun Yat-Sen University, Guangzhou 510055, China; chengyq7@mail2.sysu.edu.cn (Y.C.); jiayilin0930@163.com (J.L.); limeng48@mail2.sysu.edu.cn (M.L.); zhn16801580@163.com (H.Z.); zhuhch8@mail2.sysu.edu.cn (H.Z.); chenghh3@mail.sysu.edu.cn (H.C.); 18922102912@163.com (Q.H.); yingjieliu19@gmail.com (Y.L.); 2 Department of Shoulder and Elbow Surgery, Center for Orthopedic Surgery, The Third Affiliated Hospital of Southern Medical University, Guangzhou 510630, China; zhaoliang717@sina.com; 3 State Key Laboratory of Ophthalmology, Guangdong Provincial Key Laboratory of Ophthalmology and Visual Science, Zhongshan Ophthalmic Center, Sun Yat-Sen University, Guangzhou 510055, China; yuhuan@gzzoc.com

**Keywords:** COVID-19, Sjögren’s syndrome keratoconjunctivitis sicca, dry eye, auto-immune disease, immune microenvironment

## Abstract

**Background:** Although autoimmune complications of COVID-19 have aroused concerns, there is no consensus on its ocular complications. Sjögren’s syndrome is an autoimmune disease accompanied by the ocular abnormality keratoconjunctivitis sicca (SS-KCS), which may be influenced by COVID-19. Thereby, we explored the possible interaction between COVID-19 and SS-KCS, and we aimed to elucidate the potential correlated mechanism. **Methods:** Differentially expressed genes (DEGs) in COVID-19 and SS-KCS transcriptome data obtained from the gene expression omnibus database were identified, and COVID-19-related genes were screened using weighted gene coexpression network analysis. Common genes were verified using four machine-learning diagnostic predictors. The clinical relationship between the two common hub genes of COVID-19 was analyzed. Finally, the immune cell types infiltrating the microenvironment in the COVID-19 dataset were analyzed using CIBERSORT, and the interrelation between key genes and differentially infiltrating immune cells was verified via Pearson correlation. **Results:** Ten potential primary hub mRNAs were screened by intersecting the COVID-19 DEGs, SS-KCS DEGs, and WGCNA genes. After a multifaceted evaluation using four mainstream machine-learning diagnostic predictors, the most accurate and sensitive random forest model identified CR1 and TAP2 as the common hub genes of COVID-19 and SS-KCS. Together with the clinical information on COVID-19, the expression of CR1 and TAP2 was significantly correlated with the status and severity of COVID-19. CR1 and TAP2 were significantly positively correlated with M0 and M2 macrophages, neutrophils, and CD4+ memory resting T cells and negatively correlated with activated NK cells, monocytes, and CD8+ T cells. **Conclusions:** We validated the hub genes associated with both COVID-19 and SS-KCS, and we investigated the immune mechanisms underlying their interaction, which may help in the early prediction, identification, diagnosis, and management of SARS-CoV-2 infection-related SS-KCS syndrome or many other immune-related complications in the long COVID period.

## 1. Introduction

The novel coronavirus disease 2019 (COVID-19) has exposed the world to a global public health crisis. The damage caused by SARS-CoV-2, the etiological agent of COVID-19, arises not only from the direct infection of host cells through binding to angiotensin-converting enzyme 2 (ACE2) receptors [1] but also from systemic immune dysregulation and hyperinflammatory responses [2,3]. COVID-19-induced immune dysfunction involves both hyperactivation and immune exhaustion [4,5], increasing the risk of various complications in infected patients. In addition to antiviral strategies, restoring and modulating immune homeostasis is crucial for treating COVID-19 and preventing medium- and long-term complications [6,7].

Sjögren’s syndrome is a chronic inflammatory autoimmune disease characterized by the inflammation of exocrine glands, particularly the salivary and lacrimal glands [8]. In Sjögren’s syndrome, the lacrimal glands are infiltrated by immune cells, including CD4+ helper T cells, CD8+ cytotoxic T cells, B cells, plasma cells, macrophages, dendritic cells, and mast cells, leading to reduced tear production and dry eyes, a condition referred to as Sjögren’s syndrome keratoconjunctivitis sicca (SS-KCS) [9]. Given the acute inflammatory response and immune dysfunction during SARS-CoV-2 infection, researchers have started exploring the shared mechanisms between COVID-19 and Sjögren’s syndrome. An increasing number of studies have reported the occurrence of various autoimmune diseases during or after COVID-19. A large-scale population-based study confirmed that a COVID-19 cohort exhibited a significantly higher risk of developing autoimmune diseases, including rheumatoid arthritis, ankylosing spondylitis, systemic lupus erythematosus, dermatopolymyositis, systemic sclerosis, and Sjögren’s syndrome [10]. Additionally, another study specifically reported an increased incidence of Sjögren’s syndrome following COVID-19 [11]. However, whether different COVID-19-induced autoimmune diseases share the same mechanism and whether a specific link exists between COVID-19 and Sjögren’s syndrome remain unclear. Identifying shared immune mechanisms between COVID-19 and Sjögren’s syndrome could help in recognizing autoimmune manifestations in post-COVID-19 patients, facilitating earlier diagnosis and intervention for conditions such as SS-KCS. Given that the ocular surface is a primary site of involvement in Sjögren’s syndrome, clarifying the relationship between SS-KCS and COVID-19 is of great significance for understanding the ocular complications in SARS-CoV-2 infection, which would help in distinguishing COVID-19-induced SS-KCS from primary dry eye, improving differential diagnoses and guiding individualized immunotherapy. At the same time, it would help us break through the traditional conception of ocular diseases caused by direct viral infection of the eye and bring COVID-19-related ocular diseases into the perspective of broader immune disorders.

With advances in gene sequencing technologies and the rise of open data-sharing platforms, an increasing number of studies have integrated mass data across various diseases to explore novel mechanisms and markers using bioinformatics methods. Weighted gene coexpression network analysis (WGCNA) is a method of clustering genes according to expression patterns, systematically analyzing the relationship between gene modules and traits, and classifying gene functions. It has been widely used in bioinformatics research on prognostic markers and therapeutic targets of many diseases. Various machine-learning algorithms have been extensively utilized to predict new biomarkers and uncover novel insights into disease pathogenesis. They not only offer an unbiased approach to predicting patients’ clinical status but also enable the detection of previously unknown conditions and the identification of novel biomarkers [12]. Many studies have examined the interaction between COVID-19 and autoimmune diseases using machine learning and other bioinformatics approaches [13,14,15]. For example, Luo et al. identified potential common pathogenic mechanisms between COVID-19 and Sjögren’s syndrome by analyzing the gene expression omnibus (GEO) dataset, highlighting 12 key hub genes as potential diagnostic markers for both conditions [16]. However, the correlation between the ocular manifestations of Sjögren’s syndrome and COVID-19 remains unexplored.

In this study, we investigated the biological relationship between COVID-19 and SS-KCS. Transcriptome sequencing data of COVID-19 and SS-KCS were obtained from the GEO dataset to identify differentially expressed genes (DEGs), while COVID-19-related mRNAs were screened using WGCNA. The molecular and cellular intersections were identified using machine learning and clinical correlation analyses. Additionally, the correlation between microenvironmental immune cell components and common genes was analyzed using cell type identification by estimating the relative subsets of RNA transcripts (CIBERSORT). Our findings provide insights into the association between COVID-19 and ocular diseases, contributing to improved prevention, diagnosis, and treatment strategies for SARS-CoV-2-related SS-KCS and dry eye disease (DED).

## 2. Materials and Methods

### 2.1. Datasets and Clinical Profiles of COVID-19 and SS-KCS

The COVID-19 expression array, GSE164805, downloaded from the GEO database, contained lncRNA data, mRNA data, and clinical information for 10 COVID samples and 5 healthy control samples [17]. The SS-KCS mRNA expression array, GSE176510, contained 7 SS-KCS and 19 normal control samples [18].

### 2.2. Identification of Common DEGs Between COVID-19 and SS-KCS

In the first step of our analysis, the mRNA transcriptome data of COVID-19 array was extracted separately from lncRNA data in GSE164805 using perl based on the annotation of the GPL26963-20921 platform (Supplementary Table S2). The mRNA data of COVID-19 and SS-KCS array were normalized using the “normalizeBetweenArrays” function in the “limma” R package. The DEGs between COVID-19 and healthy samples were identified with |log2FC| > 1.0 and an adjusted *p* value < 0.05, which was considered statistically significant. The DEGs between SS-KCS and control samples were identified with |log2FC| > 0.5 and *p* values < 0.05, which were considered statistically significant. The “pheatmap” R packages were used to draw heatmap of the DEGs. A Venn diagram was used to determine the intersection of the DEGs in COVID-19 and SS-KCS samples.

### 2.3. Functional Annotation of Common DEGs

The “clusterProfiler” R package was used to perform gene ontology (GO) enrichment analysis of common DEGs to identify the biological processes (BPs), cellular components (CCs), and molecular functions (MFs) involved in COVID-19 and SS-KCS simultaneously. The *Kyoto Encyclopedia of Genes and Genomes* (KEGG) analysis was used to annotate the signaling pathways in which the DEGs were enriched, reflecting the alterations in signaling pathways in both COVID-19 and SS-KCS. The “ggplot2” package was used to draw the bubble plot, and an adjusted *p* value < 0.05 was considered statistically significant.

### 2.4. Construction of WGCNA Modules and Identification of COVID-19-Related Genes

WGCNA identifies gene coexpression networks based on topological overlaps. Highly interconnected genes are grouped into modules using hierarchical clustering with hierarchical clustering based on the connectivity and covariation coefficients of the genes. Eigengene expression patterns within each module are integrated into a “Module eigengene (ME)”. MEs in the same cluster exhibit high correlations, consistent expression patterns, and similar biological functions, which are helpful for further exploring the functions of different clusters [19]. After excluding mRNAs in the COVID-19 array with an average expression of less than 1, genes with the top 25% variance were subjected to WGCNA [20]. The power value is a critical soft-thresholding parameter used to enhance strong positive correlations between genes within the same module. The “WGCNA” R package was employed to test the independence and average connectivity of different modules under different power values, and the power value corresponding to an independence index, R^2^ = 0.8, was selected. The minimum number of genes in the mRNA modules was set to 30 [21]. Thousands of mRNAs were clustered into different modules (identified using arbitrary colors). Based on clinical information, the correlation between modules and clinical phenotypes was calculated, and modules related to clinical characteristics, such as age, sex, and COVID-19 state and stage, were screened to obtain COVID-19-related genes with *p* < 0.05 as a significance threshold.

### 2.5. Identification of Hub Common Genes via Machine Learning

After taking the intersection of common DEGs and COVID-19-related genes, the common genes were screened using four machine-learning algorithms: random forest (RF), support vector machine (SVM), extreme gradient boosting (XGB), and generalized linear model (GLM) in the COVID-19 and SS-KCS datasets. Model performance was evaluated based on residuals and the area under the curve (AUC), and the best-performing model was selected. Key genes were identified by assessing their importance in the optimal model, and most hub genes were determined by examining the intersection of key genes in the COVID-19 and SS-KCS datasets.

### 2.6. Validation of the Clinical Relevance of Hub Genes

The expression of hub genes was verified, and their correlation was determined. The expression of hub genes among groups with different clinical traits was analyzed using Student’s *t*-test or Wilcoxon test. The “ggplot2” R package was used to generate boxplots.

### 2.7. Analysis of Microenvironmental Immune Cells in COVID-19 and SS-KCS

The CIBERSORT algorithm can identify the abundance and proportion of different cell types in mixed cell populations. By analyzing the mRNA expression profiles, CIBERSORT can easily recognize each cell type and its count in the given sample [22]. In this study, we used the CIBERSORT algorithm to identify the proportions of 22 immune cell types infiltrating the microenvironment of COVID-19, SS-KCS, and normal samples. To enhance the result reliability, the analysis was repeated 100 times. The “limma” package was used to verify the differences in the proportion of immune cells between disease and healthy control, and a boxplot and bar plot were drawn using the R software (v.4.1.2).

### 2.8. Analysis of Immune Crosstalk with Hub Genes and Immune Cells

The correlation among immune cells and hub genes in the COVID-19 microenvironment was determined using the Spearman correlation with the “corrplot” package. A correlation heat map and circle graph were generated. Furthermore, the correlation between hub genes and each type of immune cell was verified individually, and correlation dot plots were generated.

### 2.9. Statistical Analysis

The R software (v.4.1.2) and the aforementioned packages were used to perform all statistical analyses and mapping. The DEGs between the two groups were identified using the Student’s t-test, whereas Spearman correlation was used to evaluate the correlation between hub genes and immune cells. Differences were considered statistically significant at *p* < 0.05 (*), *p* < 0.01 (**), and *p* < 0.001 (***).

**Figure 1:**
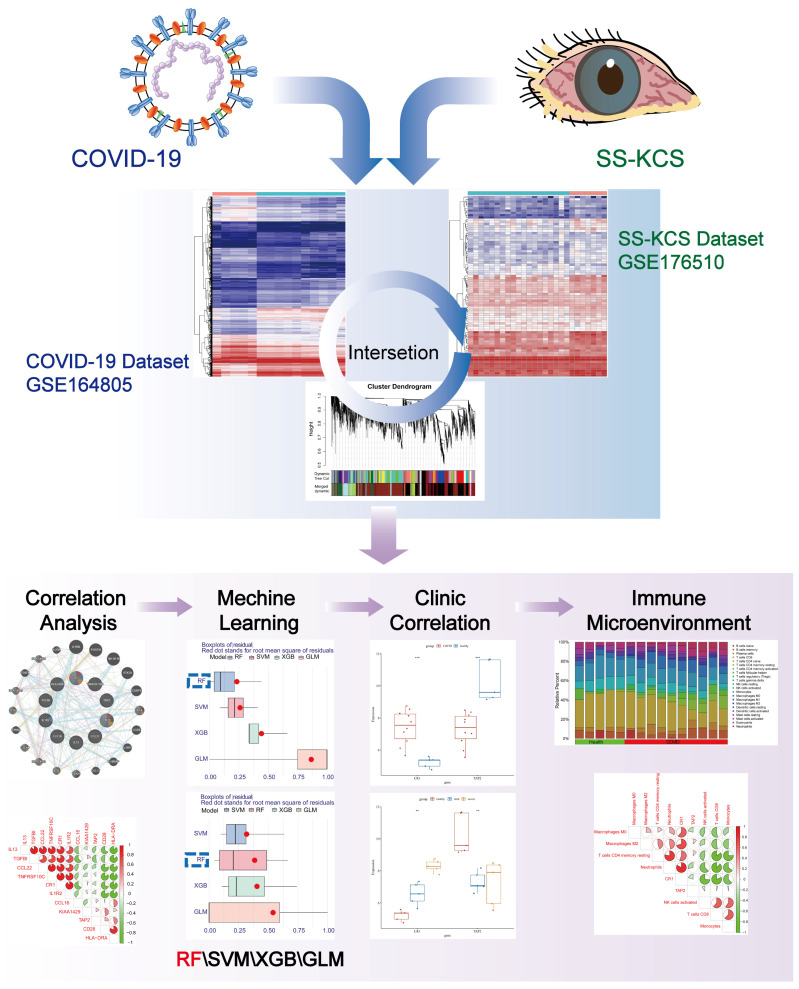
Flow chart of analyses performed in this study. ** *p* < 0.01; *** *p* < 0.001.

The general design of the present study is presented in Figure 1.

## 3. Results

### 3.1. Preliminary Screening and Annotation of COVID-19- and SS-KCS-Related Genes

**Figure 2:**
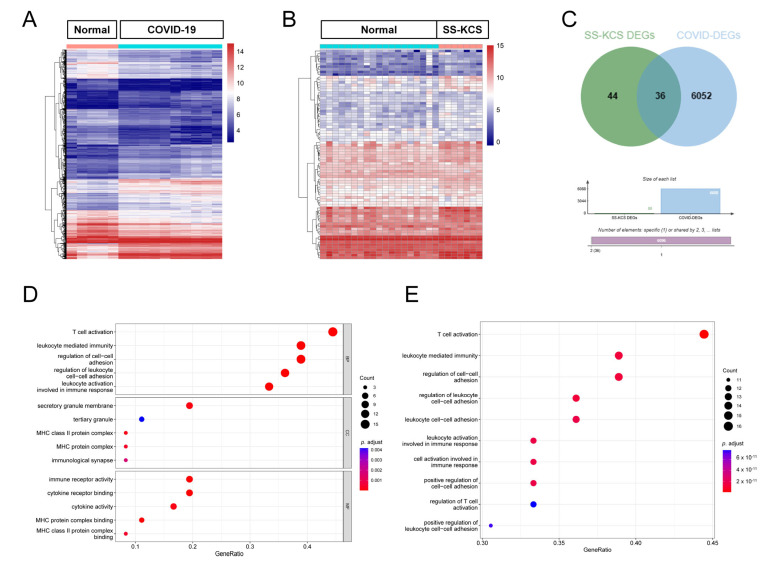
Detection and functional assessment of DEmRNAs. (**A**) Heatmap of COVID-19 DEmRNAs analyzed from the gene expression omnibus (GEO) GSE164805 dataset. Higher expression is represented by red, while blue indicates lower expression. (**B**) Heatmap of SS-KCS DEmRNAs analyzed from the GEO GSE176510 database. (**C**) Venn diagram showing the intersection of SS-KCS and COVID-19 DEmRNAs, with 36 common genes. (**D**) Gene ontology (GO) function annotation of 36 common DEmRNAs. More pronounced enrichment of specific functions is denoted by larger bubbles and taller columns. Smaller *p* values are represented by blue, while larger *p* values are represented by red. (**E**) KEGG functional enrichment of 36 overlapping DEmRNAs.

A total of 6088 differentially expressed mRNAs (DEmRNAs) were identified between COVID-19 and healthy control samples using the “limma” package (Figure 2A), while 80 DEmRNAs were detected between SS-KCS and normal control samples (Figure 2B). To preliminarily screen mRNAs correlated with both COVID-19 and SS-KCS simultaneously, we intersected the DEGs from the two disease datasets and identified 36 common DEGs (Figure 2C). The 36 common genes identified through the Venn diagram analysis showed a significant intersection of DEGs between COVID-19 and SS-KCS. These genes are of particular interest because they highlight the shared molecular mechanisms and pathways involved in both diseases, which would be helpful in understanding the shared pathophysiological mechanisms and potential biomarkers for disease prediction.

The GO enrichment analysis of the common DEGs showed that they were mainly enriched in biological processes, such as T-cell activation (GO:0042110), leukocyte-mediated immunity (GO:0002443), the regulation of cell–cell adhesion (GO:0022407), and leukocyte activation involved in immune response (GO:0002366) (Figure 2D). The KEGG pathway enrichment analysis showed that the common DEGs were significantly involved in multiple immunological processes (Figure 2E, Supplementary Table S1).

### 3.2. Identification of COVID-19-Related Genes Through the Construction of mRNA Coexpression Modules

**Figure 3:**
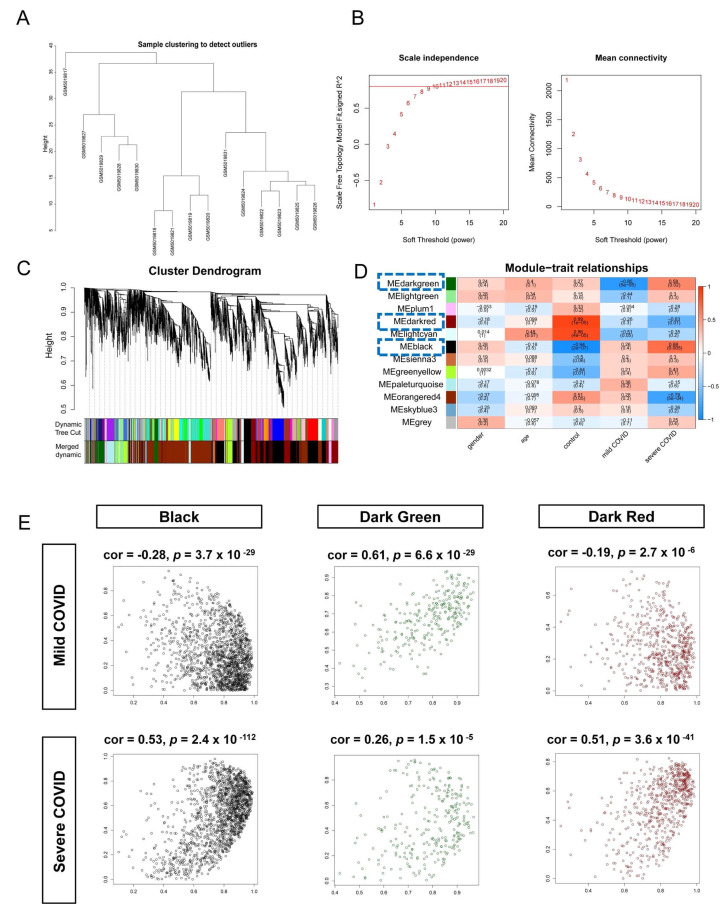
Identification of mRNAs associated with COVID-19 through WGCNA. (**A**) Hierarchical clustering tree of 15 COVID-19 sample mRNA expression patterns. (**B**) Selection of the power value through WGCNA: A power value of 14 was chosen, achieving an R^2^ > 0.8 with an average connectivity of <100. (**C**) Formation of coexpression modules through clustering and merging of mRNAs. (**D**) Heatmap visualization of the correlation between module genes and clinical characteristics. Positive correlation is indicated by red, while negative correlation is represented by blue. The intensity of the color corresponds to the strength of the correlation. (**E**) Presentation of the correlation between modules and clinical traits using a scatterplot. Modules in black, dark green, and dark red are positively associated with both mild and severe cases of COVID-19.

To enhance the robustness of the WGCNA coexpression network, the mRNA expression data of the COVID-19 dataset was normalized and filtered to remove low expression or low variance genes. Then, we constructed a WGCNA coexpression module for 20179 mRNAs from 15 samples. The “WGCNA” R package calculated the correlation matrix between all gene pairs, converted the correlation matrix to an adjacency matrix using a power function (soft thresholding). When the power value was 14, the degree of independence was >0.8, which better reflected the scale-free topology of the coexpression network (Figure 3B). Then, the hierarchical clustering tree was constructed based on the expression patterns of mRNAs derived from the COVID-19 dataset. Branches in the tree were determined using average linkage hierarchical clustering, calculated based on the Euclidean distances between gene expression profiles. The branching in the tree reflects the similarity in expression patterns between different samples (Figure 3A). The similarity between mRNAs was calculated. The dynamic cut algorithm ensures that the similarity between genes within each module is high enough, while the differences between genes in different modules are significant. Twelve mRNA coexpression modules were identified. The number of eigengenes contained in the modules ranged from a minimum of 60 (gray modules) to a maximum of 1625 (orangered 4 modules, Figure 3C).

Using the clinical information from the GSE164805 dataset, the correlation between the gene modules and clinical characteristics was analyzed (Figure 3D). The dark green, dark red, and black modules exhibited a significant correlation with the COVID-19 status (*p* < 0.05, Figure 3E). Therefore, the preliminary analysis identified 2413 mRNAs (COVID-WGCNA) within these modules as potential contributors to COVID-19 prognosis.

### 3.3. Screening for Common Hub Genes Correlated with COVID-19 and SS-KCS

**Figure 4:**
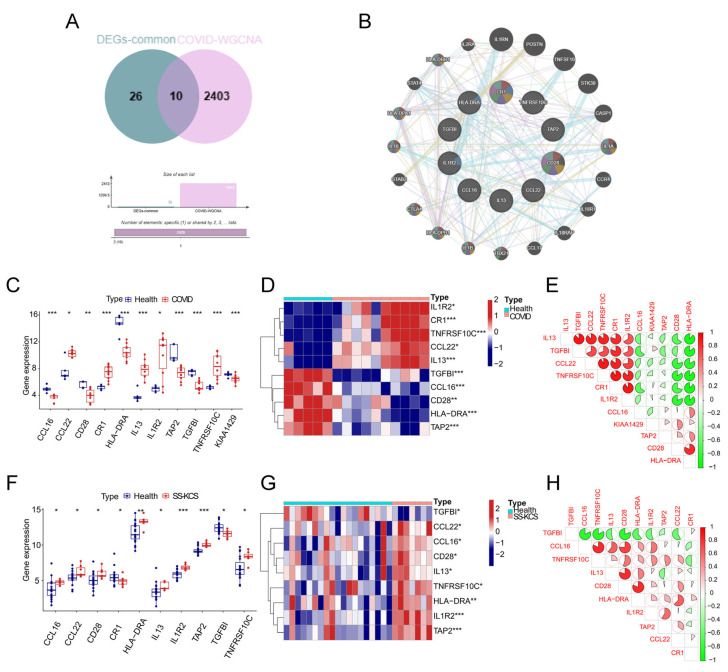
Preliminary recognition of genes shared between COVID-19 and SS-KCS. (**A**) The intersection of common DEGs and COVID-19-WGCNA-mRNAs yielded 10 potential hub mRNAs. (**B**) A gene interaction network was constructed based on these 10 hub genes using the GeneMANIA database. The outer circle highlights the top 20 closely associated neighboring genes. (**C**) Comparison of the expression of 10 potential hub genes between COVID-19 and healthy controls. (**D**) Expression heatmap of the 10 potential hub genes in the COVID-19 dataset. (**E**) Correlation heatmap of the 10 potential hub genes in the COVID-19 dataset. (**F**) Comparison of the expression of the 10 potential hub genes between SS-KCS and health controls. (**G**) Expression heatmap of 10 potential hub genes in the SS-KCS dataset. (**H**) Correlation heatmap of the 10 potential hub genes in the SS-KCS dataset. * *p* < 0.05; ** *p* < 0.01; *** *p* < 0.001.

To enhance the disease specificity of hub genes, we examined the intersection of 36 common DEGs and 2413 COVID-WGCNA genes. Ten intersecting genes (TGFBI, TAP2, IL1R2, CR1, TNFRSF10C, CD28, CCL16, HLA-DRA, CCL22, and IL13) were identified (Figure 4A), and a gene interaction network was established using the GeneMANIA database (Figure 4B). The expression levels of these 10 genes were analyzed in both the COVID-19 and SS-KCS datasets. The boxplots show that the 10 genes were significantly differentially expressed between the two diseases and normal controls (Figure 4C,F). Heatmaps of the 10 genes were generated for both the COVID-19 (Figure 4D) and SS-KCS (Figure 4G) datasets. The correlation of the 10 genes in the COVID-19 (Figure 4E) and SS-KCS (Figure 4H) datasets was analyzed, and correlation heatmaps were generated.

**Figure 5:**
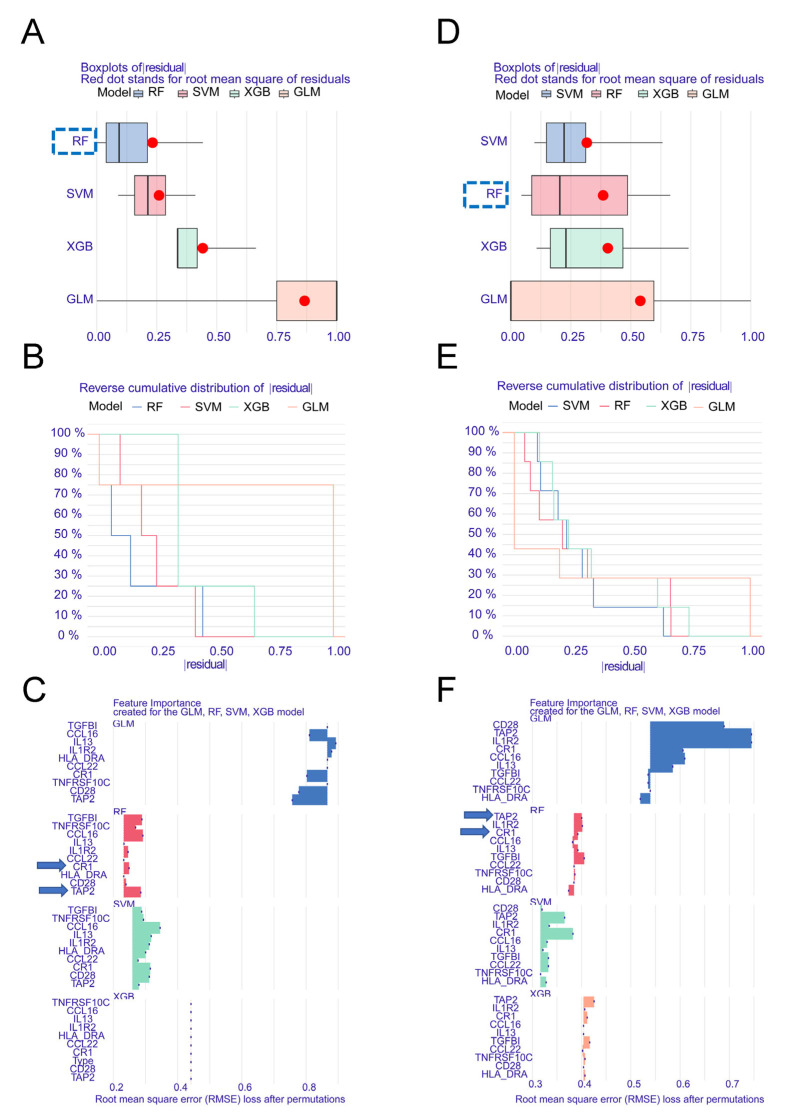
Multifaceted evaluation of four mainstream machine-learning diagnostic predictors in COVID-19 and SS-KCS. (**A**) Machine-learning models, including RF, SVM, XGB, and GLM were constructed using 10 potential hub genes in the COVID-19 dataset. The boxplot of residuals of the four models was generated. The smaller residuals indicated higher accuracy. (**B**) Reverse cumulative distribution of residuals across the four models in the COVID-19 dataset. (**C**) Determining the significance of pivotal genes in the four models; the top 4 hub genes in the COVID-19 dataset were CR1, TNFRSF10C, TAP2, and TGFBI. (**D**) The boxplot of residuals of the four models in the SS-KCS dataset. (**E**) Reverse cumulative distribution of residuals across the four models in the SS-KCS dataset. (**F**) The top 4 hub genes in the SS-KCS dataset were CR1, IL13, TAP2, and IL1R2. Among them, CR1 and TAP2 were shared between the two diseases, suggesting their potential role as common diagnostic markers.

To identify hub genes involved in the interaction between COVID-19 and SS-KCS, four machine-learning algorithms were employed to validate the 10 common genes. Based on the residual parameters, the RF model demonstrated the highest specificity and accuracy in both the COVID-19 (Figure 5A,B) and SS-KCS (Figure 5D,E) datasets. Further analysis of gene importance across the four models revealed that the top four hub genes in COVID-19 were CR1, TNFRSF10C, TAP2, and TGFBI (Figure 5C), while the top four hub genes in SS-KCS were CR1, IL13, TAP2, and IL1R2 (Figure 5F). The intersection of the top-ranked genes in the two diseases identified CR1 and TAP2 as shared hub genes, suggesting their potential role in linking COVID-19 and SS-KCS.

### 3.4. Verification of Clinical Correlation of CR1 and TAP2 in the COVID-19 Dataset

**Figure 6:**
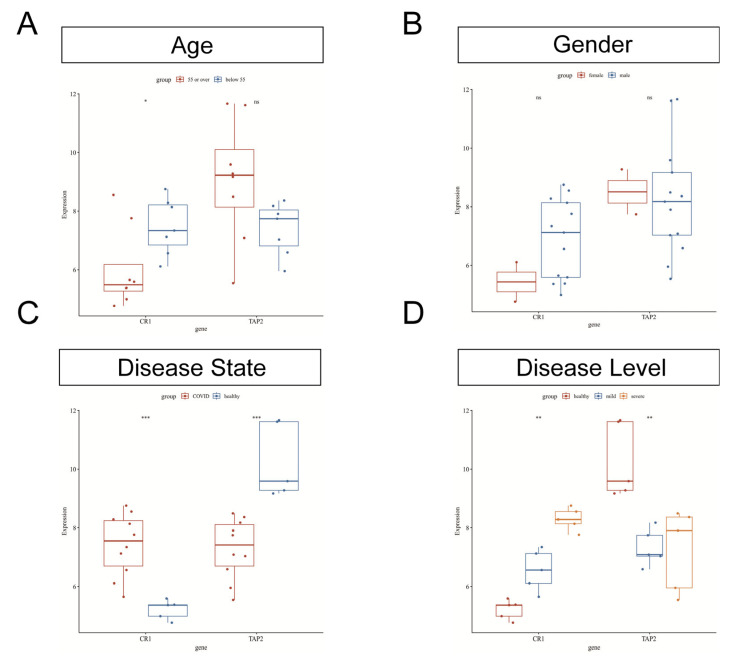
Clinical correlation of CR1 and TAP2 in the COVID-19 dataset. (**A**) R1 expression was lower in patients aged >55 years, while TAP2 showed no significant correlation with age. (**B**) The expression of CR1 and TAP2 have no significant differences between male and female patients. (**C**) The expression of CR1 was higher in COVID-19 patients whereas that of TAP2 was higher in healthy people. (**D**) With regard to the severity of COVID-19, expression patterns of CR1 and TAP2 varied significantly across different COVID-19 severity levels. * *p* < 0.05; ** *p* < 0.01; *** *p* < 0.001; ns: no statistical significance.

The COVID-19 dataset contained the clinical information, including age, sex, and disease severity, for 10 patients. The expression levels of CR1 and TAP2 were identified across various clinical groups. The expression of CR1 was higher in patients under 55 years of age, and TAP2 expression showed no correlation with age (Figure 6A). No significant sex-based differences were observed for both genes (Figure 6B). Regarding the status and severity of COVID-19, CR1 was significantly upregulated in COVID-19 patients, whereas TAP2 exhibited higher expression in healthy individuals (Figure 6C). C Expression patterns varied across disease severity levels: CR1 showed the highest expression in severe COVID-19 cases and the lowest in healthy individuals, while TAP2 was most highly expressed in healthy samples and lowest in mild COVID-19 cases (Figure 6D).

### 3.5. Qualification of Immune Cell Infiltration Within the Microenvironment of COVID-19 and SS-KCS

**Figure 7:**
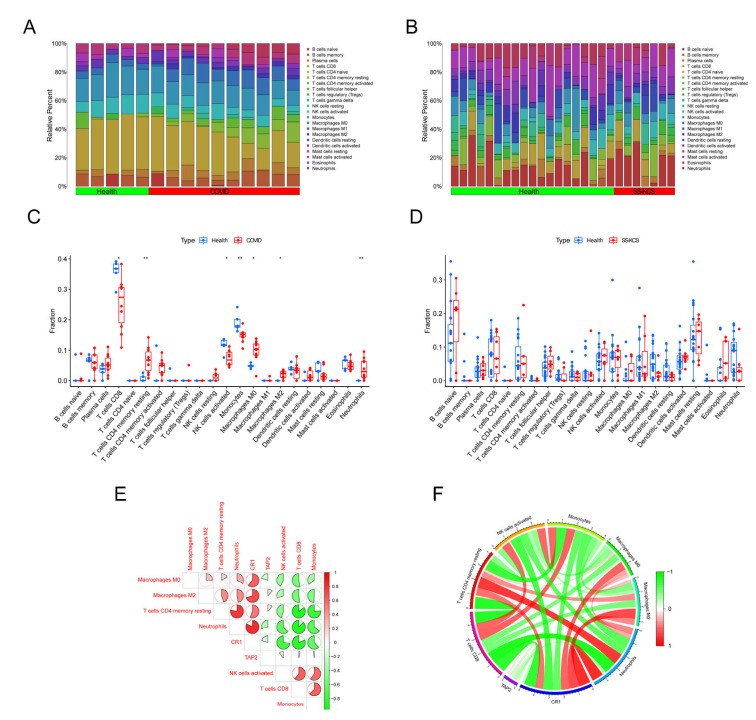
Analysis of immune cells in the microenvironment in the COVID-19 and SS-KCS datasets. Graphs illustrating the distribution of 22 immune cell types within the microenvironment across various samples in the COVID-19 (**A**) and SS-KCS (**B**) datasets. (**C**) Comparison of immune cell infiltration proportions between COVID-19 and healthy samples. Significant differences were observed in the proportions of CD8+ T cells, CD4+ memory resting T cells, activated NK cells, monocytes, M0 and M2 macrophages, and neutrophils between COVID-19 and healthy controls (all *p* < 0.05). (**D**) Comparison of the proportions of the 22 immune cell types infiltrating the microenvironment in SS-KCS and healthy controls. No significant differences were observed. (**E**) Heatmap illustrating the correlations among immune cells that exhibited significant differences in the COVID-19 dataset. (**F**) Correlation circle diagram depicting relationships among significantly different immune cells in the COVID-19 dataset. * *p* < 0.05; ** *p* < 0.01.

We employed the CIBERSORT algorithm to assess the composition of 22 immune cell subtypes in the microenvironments of COVID-19 and SS-KCS. The proportions of the 22 immune cells in each COVID-19 (Figure 7A) and SS-KCS (Figure 7B) sample are presented as bar plots. Differences in immune cell infiltration between disease and healthy samples were analyzed (Figure 7C,D). The proportions of CD8+ T cells, CD4+ memory resting T cells, activated NK cells, monocytes, M0 and M2 macrophages, and neutrophils were significantly different between COVID-19 and healthy samples (all *p* < 0.05). Additionally, correlations among immune cell types with differential proportions in the COVID-19 dataset were analyzed (Figure 7E,F).

### 3.6. Correlation Between CR1, TAP2, and the Microenvironmental Immune Cells

**Figure 8:**
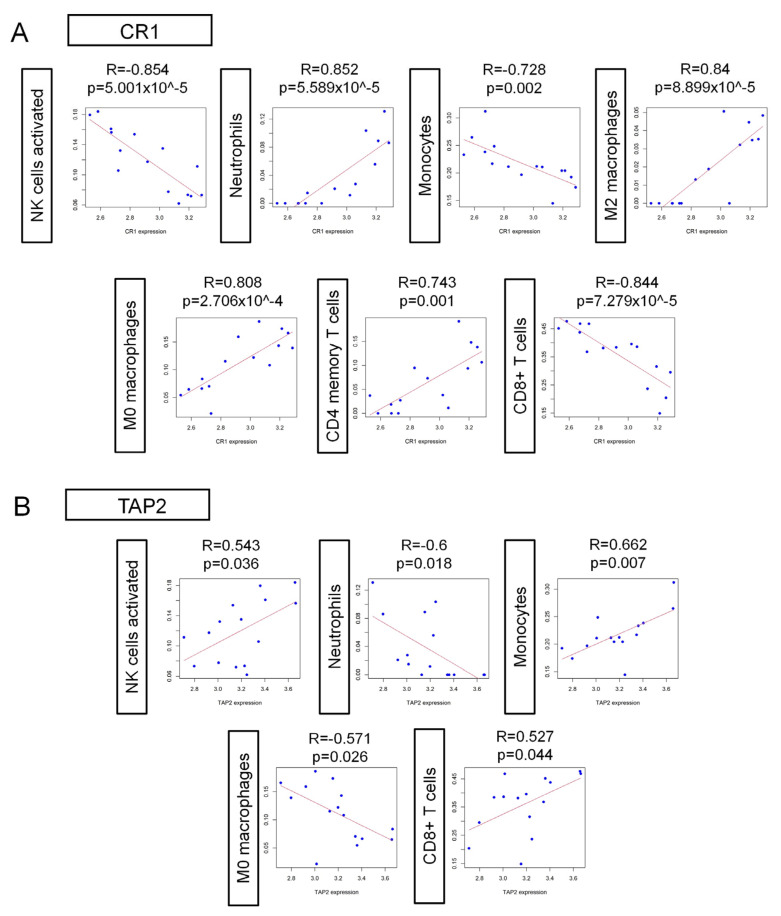
Correlation analysis of CR1 (**A**) and TAP2 (**B**) with immune cell composition in the microenvironment. All *p* values < 0.05.

To further elucidate the role of hub genes in the interaction between COVID-19 and SS-KCS, we performed a Pearson correlation analysis to assess the correlation between CR1, TAP2, and various immune cells. The analysis was conducted using the expression levels of CR1 and TAP2, as well as the infiltration proportions of immune cells, as input parameters. Statistical significance was assessed using the Benjamini–Hochberg procedure to control the false discovery rate (FDR < 0.05). A correlation analysis revealed that CR1 was significantly positively correlated with neutrophils, M0 and M2 macrophages, and CD4 memory resting T cells, while it was negatively correlated with activated NK cells, monocytes, and CD8+ T cells (all *p* < 0.05; Figure 8A). TAP2 exhibited an opposite correlation pattern to CR1 (all *p* values < 0.05; Figure 8B). The correlation coefficients of each analysis are shown in corresponding panels.

## 4. Discussion

The COVID-19 outbreak has induced an extremely increased prevalence of autoimmune diseases, suggesting that the medium- and long-term complications of COVID-19 are not only caused by direct SARS-CoV-2 infection but also result from systemic inflammation affecting multiple tissues and organs [23,24,25]. Most studies attribute COVID-19-related DED to lifestyle changes [26,27,28]. Considering the important roles played by immune disorders in DED, we speculated that COVID-19 promotes the progression of DED via immunological approaches. Dry eye syndrome is one of the primary ocular manifestations in patients with SS-KCS. In this study, we identified, for the first time, a shared molecular mechanism between COVID-19 and SS-KCS. We combined DEG analysis and WCGNA to identify the common hub genes linking the two diseases and developed a robust predictive model using four mainstream machine-learning algorithms to estimate the risk of SS-KCS in patients with COVID-19. This approach significantly improved the accuracy of biomarker prediction while ensuring a strong correlation between SS-KCS and COVID-19. We verified the correlation of the common hub genes with the clinical value and immune cell infiltration of the microenvironment. This approach significantly improved the accuracy of biomarker prediction while ensuring a strong correlation between SS-KCS and COVID-19.

WGCNA constructs coexpression networks by calculating the expression similarity between genes and identifies gene modules with a high degree of coexpression. It is widely used to explore gene clusters associated with specific physiological processes or diseases, identify key genes that may regulate entire modules, build more comprehensive gene regulatory networks, uncover associations between gene expression data and clinical data, and provide potential biological markers. WGCNA has been used to identify the hub genes in Sjögren’s syndrome or COVID-19 in previous studies [29,30,31]. For instance, Lai et al. analyzed mRNA expression profiles of COVID-19 from the GEO database, identified SARS-CoV-2 infection-related mRNA coexpression modules through WGCNA, and screened three hub mRNAs (CLEC4D, DUSP13, and UNC5A) correlated with immune activities through mechanism learning [31]. Several studies have also explored the interactions between COVID-19 and multiple system diseases. Zhang et al. identified the shared molecular signatures of SARS-CoV-2 infection and its influence on acute kidney injury and chronic kidney disease, and they found four hub-interacting genes (DUSP6, BHLHE40, RASGRP1, and TAB2) [32]. In another study, the impact of SARS-CoV-2 infection on inflammatory bowel disease was analyzed by mining GEO RNA-sequencing and single-cell RNA-sequencing datasets, highlighting an increased proportion of CD14+ monocytes in COVID-19 patients and validating 38 hub genes related to CD14+ monocytes [13]. This work is the first to investigate the interaction of ocular diseases and COVID-19 using bioinformatics methods. First, we systematically classified gene expression modules and DEGs to screen the common DEGs between the two diseases (Figure 2, Figure 3 and Figure 4). Next, multiple mainstream machine-learning algorithms were employed to further identify four hub genes (Figure 5). By integrating clinical characteristics (Figure 6) with the immune cell composition of the COVID-19 microenvironment (Figure 7 and Figure 8), we further validated the hub genes associated with the immune microenvironment in both diseases.

The two hub mRNAs, CR1 and TAP2, play important roles in the development of Sjögren’s syndrome and other autoimmune diseases while also contributing to COVID-19 progression. CR1 encodes a type I transmembrane glycoprotein that functions as a complement regulatory protein with multiple immunological roles [33]. Reduced CR1 levels have been observed in patients with autoimmune diseases, such as systemic lupus erythematosus, rheumatoid arthritis, Sjögren’s syndrome, and paracoccidioidomycosis, potentially altering the binding site of the immune complex [34]. In a study on CR1 levels in patients with primary Sjögren’s syndrome, CR1 levels varied within a wide range (20–124%), with a notable prevalence of lower CR1 levels among affected individuals [35]. However, the CR1 levels in the ocular tissues with SS-KCS have not been determined. In our study, we performed the first explicit analysis of the CR1 expression in conjunctiva with Sjögren’s syndrome, revealing that CR1 expression was significantly higher compared to normal controls. A decrease in CR1 levels was also found in patients with COVID-19 in intensive care units, along with hyperactivation of the complement system, which contributes to immune disorders during SARS-CoV-2 infection [36]. However, several previous studies have reported contrasting findings regarding CR1 expression in COVID-19 patients compared to our results. We speculate that COVID-19 is a progressive disease with different, even opposite, pathological processes at different stages. In the early stages of COVID-19, the immune response is delayed, and the immune refractory period is prolonged. In the late stages of COVID-19, many inflammatory responses are activated, resulting in hyperinflammation [37]. These dynamic shifts in immune status and CR1-mediated complement regulation may vary over time, underscoring the need for larger sample analyses and further investigation into the underlying mechanisms.

TAP2, a transporter associated with antigen processing, plays an important role in immune responses and autoimmune diseases [38]. The upregulation of TAP2 in Sjögren Syndrome has been previously reported [39], aligning with our finding in this study. In SS-KCS mouse models, TAP2 expression exhibited quantitative and temporal variations [36], indicating its important role in the progression of autoimmune diseases. Nonetheless, TAP2 expression exhibited quantitative and temporal variations

The abnormal accumulation and functioning of immune cells in the microenvironment mediate immune disorders, drug resistance, and inflammatory turbulence. In this study, we identified distinct immune cell populations in the COVID-19 microenvironment and confirmed that the hub genes were closely correlated with several immune cells. Lanza et al. found that neutrophils with low CR1 expression exhibited reduced cytolytic activity against tumor cells, suggesting a link between the CR1 expression levels and neutrophil activity. This is consistent with our finding that CR1 was positively correlated with the proportion of neutrophils (Figure 8A). TAP2 has also been reported to play a key role in the activation of CD8+ T cells by presenting exogenous antigens via macrophages [40]. This further supports its involvement in immune modulation and highlights the potential role of TAP2 in both COVID-19 and SS-KCS.

Our findings in this study may contribute to a better understanding of long-term complications following COVID-19 and provide clinical guidance for their management. In the first place, the expression patterns of CR1 and TAP2 exhibited significant variations across different severities of COVID-19, indicating that measuring their expression levels on the ocular surface, such as in tears, may serve as a potential predictor of COVID-19 prognosis in clinical settings. Secondly, in clinical practice, if samples from SS-KCS patients exhibit positive results for both genes, this may indicate that the condition is not solely an autoimmune disorder but could also be associated with a history of COVID-19 infection. Additionally, by elucidating the impact of SARS-CoV-2 on lacrimal gland function and tear film stability, this study has the potential to inform and guide protective strategies, including artificial tear supplementation, anti-inflammatory therapies, and lifestyle modifications, thereby mitigating long-term ocular damage in patients following COVID-19 infection.

Despite the insights gained from this study, the use of bioinformatics tools to fully elucidate the biological interactions between COVID-19 and SS-KCS remains an ongoing challenge. First, although the role of CR1 and TAP2 in COVID-19 and Sjögren’s syndrome has been confirmed in numerous studies, conflicting findings persist, indicating that more studies and samples are needed. Second, the available data from public databases remain limited, and they may somewhat restrict the generalizability of our analysis. Furthermore, ocular diseases may serve as a window that reflects systemic diseases. Yet, significant gaps remain in understanding COVID-19-related and other systemic eye diseases. Third, while our model demonstrates efficient performance on moderate-sized datasets (such as those used in this study), we acknowledge that scalability may become a concern with extremely large datasets. Specifically, issues related to computational resources and model interpretability could arise. Additionally, the data preprocessing, model training, and model validation stages may impose limitations on analysis efficiency. Moreover, while we confirmed the association between immune cell proportions and hub genes, we did not investigate the specific mechanisms through which CR1 and TAP2 mediate the link between COVID-19 and SS-KCS, warranting further investigation.

## 5. Conclusions

In summary, this study is the first to explore the potential interaction between COVID-19 and SS-KCS, providing further evidence for COVID-19-related ocular diseases. In this study, we combined WGCNA, mechanism learning, and CIBERSORT algorithms to identify common hub genes linking COVID-19 and SS-KCS, and we verified their association with immune cell infiltration. This approach offers a new perspective for exploring more diverse and effective COVID-19 biological markers, which could contribute to enhancing the comprehension and improving the management of SS-KCS patients affected by SARS-CoV-2.

## Data Availability

The datasets analyzed for this study can be found in the GEO (GSE164805 and GSE176510) (“https://www.ncbi.nlm.nih.gov/geo (accessed on 27 April 2023)”).

## Supplementary Materials

The following supporting information can be downloaded at https://www.mdpi.com/article/10.3390/biomedicines13030579/s1; Table S1: Results of GO enrichment analysis of DEGs-common. Table S2: Annotation file of the GPL26963-20921 platform.

## Abbreviations

The following abbreviations are used in this manuscript:

COVID-19	Novel coronavirus disease 2019
ACE2	Angiotensin-converting enzyme 2 receptors
SS-KCS	Sjögren’s syndrome keratoconjunctivitis sicca
DEG	Differentially expressed gene
CIBERSORT	Cell type identification by estimating the relative subsets of RNA transcripts
KEGG	*Kyoto Encyclopedia of Genes and Genomes*
GO	Gene ontology
SVM	Support vector machine
XGB	Extreme gradient boosting
RF	Random forest
GLM	Generalized linear model
AUC	Area under the curve
