# Supplementary material for: Insights into the Correlation and Immune Crosstalk Between COVID-19 and Sjögren’s Syndrome Keratoconjunctivitis Sicca via Weighted Gene Coexpression Network Analysis and Machine Learning
Source: Biomedicines. 2025 Feb 25;13(3):579. doi: 10.3390/biomedicines13030579 (PMC11940795; doi:10.3390/biomedicines13030579)
Supplement: Supplementary file 1 [file biomedicines-13-00579-s001.zip › biomedicines-3450578-supplementary table S1.pdf]

Table 1 Results of GO enrichment analysis of DEGs-common-

Ontology	Term	Description	Count	adj.P value
BP	GO:0042110	T cell activation	16	1.98E-13
BP	GO:1903037	regulation of leukocyte cell-cell adhesion	13	1.23E-11
BP	GO:0002443	leukocyte mediated immunity	14	1.23E-11
BP	GO:0022407	regulation of cell-cell adhesion	14	1.23E-11
BP	GO:0002366	leukocyte activation involved in immune response	12	1.64E-11
CC	GO:0030667	secretory granule membrane	7	0.000123217
CC	GO:0042613	MHC class II protein complex	3	0.000174299
CC	GO:0042611	MHC protein complex	3	0.000389067
CC	GO:0001772	immunological synapse	3	0.001640349
CC	GO:0070820	tertiary granule	4	0.004151159
MF	GO:0140375	immune receptor activity	7	1.15E-06
MF	GO:0023023	MHC protein complex binding	4	2.88E-05
MF	GO:0005126	cytokine receptor binding	7	2.88E-05
MF	GO:0005125	cytokine activity	6	0.000154023
MF	GO:0023026	MHC class II protein complex binding	3	0.000415095
